# Kidney inflammaging is promoted by CCR2^+^ macrophages and tissue-derived micro-environmental factors

**DOI:** 10.1007/s00018-020-03719-0

**Published:** 2020-12-12

**Authors:** Lise Lefèvre, Jason S. Iacovoni, Hélène Martini, Julie Bellière, Damien Maggiorani, Marianne Dutaur, Dimitri J. Marsal, Pauline Decaunes, Nathalie Pizzinat, Jeanne Mialet-Perez, Daniel Cussac, Angelo Parini, Victorine Douin-Echinard

**Affiliations:** 1 Institute of Metabolic and Cardiovascular Diseases, UMR1048, 1 av Jean Pouhlès, BP 84225, 31432 Toulouse, Cedex 4 France; 2 Paul Sabatier University, 31062 Toulouse, Cedex 9 France; 3 Rangueil Hospital, CHU, 31059 Toulouse, Cedex 9 France

**Keywords:** Aging, Kidney, CCR2, Macrophages, CD73, Mesenchymal stromal cells, Senescence, Inflammaging

## Abstract

The incidence of disorders associated with low inflammatory state, such as chronic kidney disease, increases in the elderly. The accumulation of senescent cells during aging and the senescence-associated secretory phenotype, which leads to inflammaging, is known to be deleterious and account for progressive organ dysfunction. To date, the cellular actors implicated in chronic inflammation in the kidney during aging are still not well characterized. Using the DECyt method, based on hierarchical clustering of flow cytometry data, we showed that aging was associated with significant changes in stromal cell diversity in the kidney. In particular, we identified two cell populations up-regulated with aging, the mesenchymal stromal cell subset (kMSC) expressing CD73 and the monocyte-derived Ly6C^+^ CCR2^+^ macrophage subset expressing pro-inflammatory cytokines. Aged CD73^+^ kMSCs depicted senescence associated features with low proliferation rate, increased DNA damage foci and *Ccl2* expression. Using co-cultures experiments, we showed that aged CD73^+^ kMSC promoted monocyte activation and secretion of inflammatory cytokines albeit less efficiently than young CD73^+^ kMSCs. In the context of ageing, increased frequency of CD73^+^ kMSC subpopulations could provide additional niche factors to newly recruited monocytes favoring a positive regulatory loop in response to local inflammation. Interfering with such partnership during aging could be a valuable approach to regulate kidney inflammaging and to limit the risk of developing chronic kidney disease in the elderly.

**Supplementary Information:**

The online version contains supplementary material available at 10.1007/s00018-020-03719-0.

## Introduction

Although an increase in longevity represents progress, per se, it can become a socio-economic burden and medical problem when not associated with quality of life maintenance and disability prevention. A considerable increased risk in the incidence of diseases associated with low level chronic inflammation, such as the many different chronic kidney and cardiovascular diseases, has been observed in the aging population. The aging kidney has been characterized by a progressive functional decline associated with glomerulosclerosis, tubular atrophy and an increased risk of early allograft rejection [1]. The accumulation and persistence of cells with senescence associated secretory phenotype (SASP), during aging and after tissue injury, account for the loss of tissue homeostasis leading to progressive organ dysfunction [2, 3]. Senescent cells secrete bioactive factors, pro-inflammatory cytokines, chemokines and proteases, which collectively participate in the development of chronic low-grade inflammation [4]. Pivotal studies revealed a prominent role for persistent senescent cells and the production of SASP, in particular IL-6, in the deterioration of kidney function. Indeed, ablation of senescent (p16^Ink4a^ positive) cells by gene suicide strategies [2, 5] or by forced p53-dependent apoptosis [5], reduced glomerulosclerosis and preserved the glomerular filtration rate of aged mice.

The cellular interactions involved in the installation SASP production and the related modifications of renal microenvironment with aging remain to be determined.

In this study, we aimed to characterize the role of renal stroma on kidney aging and, in particular, its contribution to SASP production. Renal stroma is constituted by non-parenchymal cells of diverse lineages such as immune cells, endothelial cells, fibroblasts, pericytes and mesenchymal stromal cells, which are known to play a major role in tissue homeostasis and in response to injury. Thanks to the DECyt method based on hierarchical clustering of flow cytometry data, we observed major modifications related to age in the composition of renal stroma. We showed that SASP related key inflammatory cytokines such as TNF-α, IL-6 and IL-1ß are mainly produced by macrophages in the stroma of aging kidney. The frequency and activation state of the CCR2^+^ Ly6C^+^ monocyte-derived macrophage subset were increased in the kidney with aging and contributed to higher pro-inflammatory cytokine production. In renal stroma, macrophages assume key functions regulating tissue homeostasis, response to sterile injury or pathogens. In the adult kidney, macrophages constitute a heterogeneous and dynamic population, with diverse developmental origins and effector functions, influenced by age and response to injury [6–9]. In pathological settings, tissue-derived signals participate to modification of the macrophage pool and their effector functions through recruitment of blood inflammatory CCR2^+^ monocytes [8, 10] and proliferation of resident macrophages [11].

In several organs, tissue-derived factors play critical roles in driving the acquisition of effector functions by monocyte-derived macrophages [12, 13]. Using co-culture experiments, we showed that secreted factors from Sca-1^+^ renal cells enhance the production of IL-6 and TNF-α by monocytes in response to pro-inflammatory stimulation. We provided evidence that aging increases frequencies of Sca-1^+^ CD73^+^ kidney mesenchymal stromal cells (kMSCs) and reduces Sca-1^+^ tubular epithelial cells which modulates the monocyte/macrophage inflammatory response to control IL-6 production. Understanding how aging alters the cross-talk between monocyte/macrophage population and other renal stromal cells will help to define new strategies to prevent the installation SASP production to decrease the incidence of diseases associated with low-level chronic inflammation such as chronic kidney disease.

## Results

### Kidney inflammaging

**Fig. 1:**
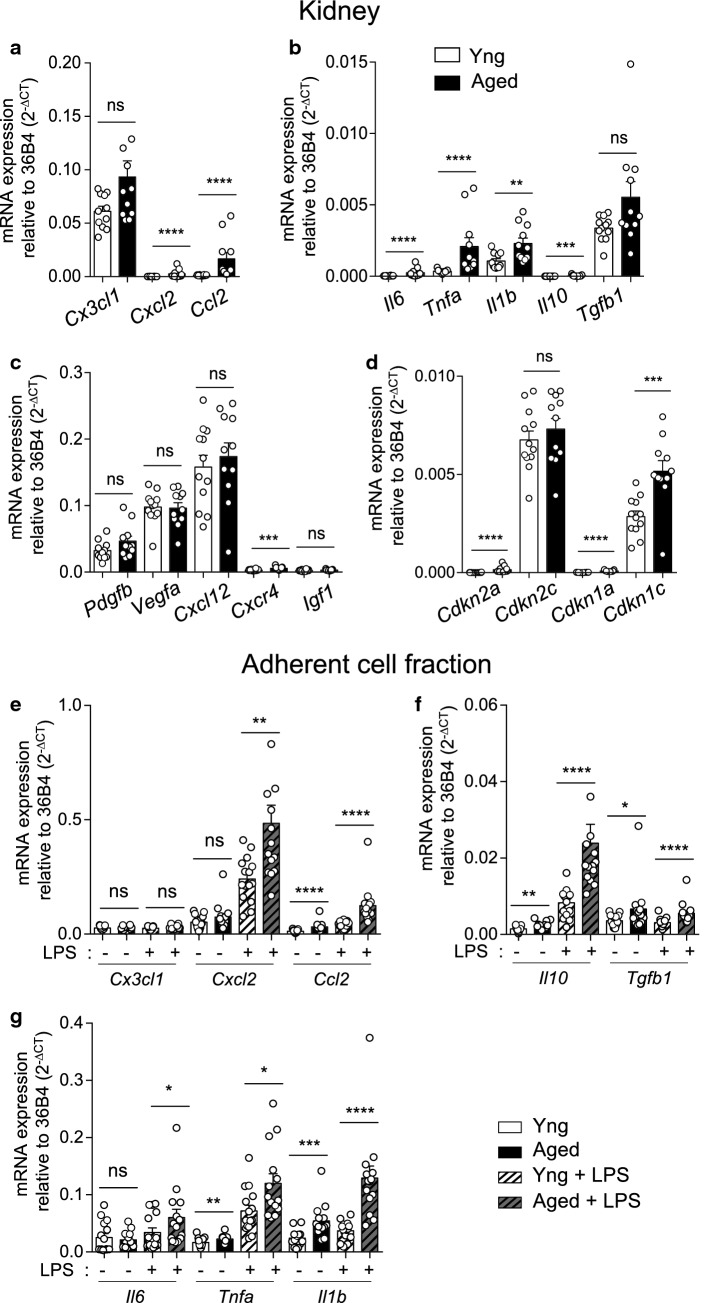
Up-regulation of pro-inflammatory mediators in the aging kidney. **a**–**d** mRNA expression of chemokines (**a**) cytokines (**b**) pro-angiogenic factors (**c**) and cyclin-dependent kinase inhibitors (**d**) by young or aged kidneys relative to house-keeping gene (*n* = 11–12 mice per group). **e**–**g** mRNA expression of chemokines (**e**), pro- (**f**) and anti- (**g**) inflammatory cytokines by adherent cells of enzymatic digested young or aged kidneys, relative to house-keeping gene, with or without LPS stimulation for 4 h (*n* = 15–14 mice per group). *P < 0.05 **P < 0.01 ***P < 0.001 ****P < 0.0001, ns, not significant, Mann–Whitney *t* test

The SASP-related factors produced during kidney aging were detected, at the tissue level, with up-regulation of mRNA expression of the chemokines *Cxcl2* and *Ccl2* (Fig. 1a), the inflammatory cytokines *Il6*, *Tnfa* and *Il1b* and the anti-inflammatory cytokines *Il10* and *Tgfb1* (Fig. 1b). Gene expression of pro-angiogenic factors, such as *Pdgfb*, *Vegfa*, *Cxcl12* and *Igf1* was not significantly increased with aging in the kidney except for *Cxcr4* (Fig. 1c). As previously reported [14], kidney aging and SASP expression were associated with the upregulation of mRNA expression of senescence associated Cdkis (*Cdkn2a*, *Cdkn1a* and *Cdkn1c*) (Fig. 1d). Concerning kidney function, although fibrosis is mildly increased with aging (Supp Fig. 1a, b), glomerular filtration is maintained with no significant increase of creatininemia and BUN in 24-month-old animals (Supp Fig. 1c).

To explore the role of renal stromal cells in kidney inflammaging, we tested mRNA expression of these immune regulating factors by the adherent cell fraction of the kidney, enriched in renal stromal cells, using LPS stimulation as positive control (Fig. 1e–g). At the steady state, aging was associated with significant up-regulation of the pro-inflammatory mediators *Ccl2*, *Tnfa* and *Il1b* and of the anti-inflammatory cytokines *Il10* and *Tgfb1* (Fig. 1e–g). Except for *Cx3cl1*, LPS-induced higher expression of these factors from aged mice compared to young (Fig. 1e–g) reflecting the installation of a pro-inflammatory activation state in the aging kidney.

### Impact of aging on renal stromal cell subsets in vivo

**Fig. 2:**
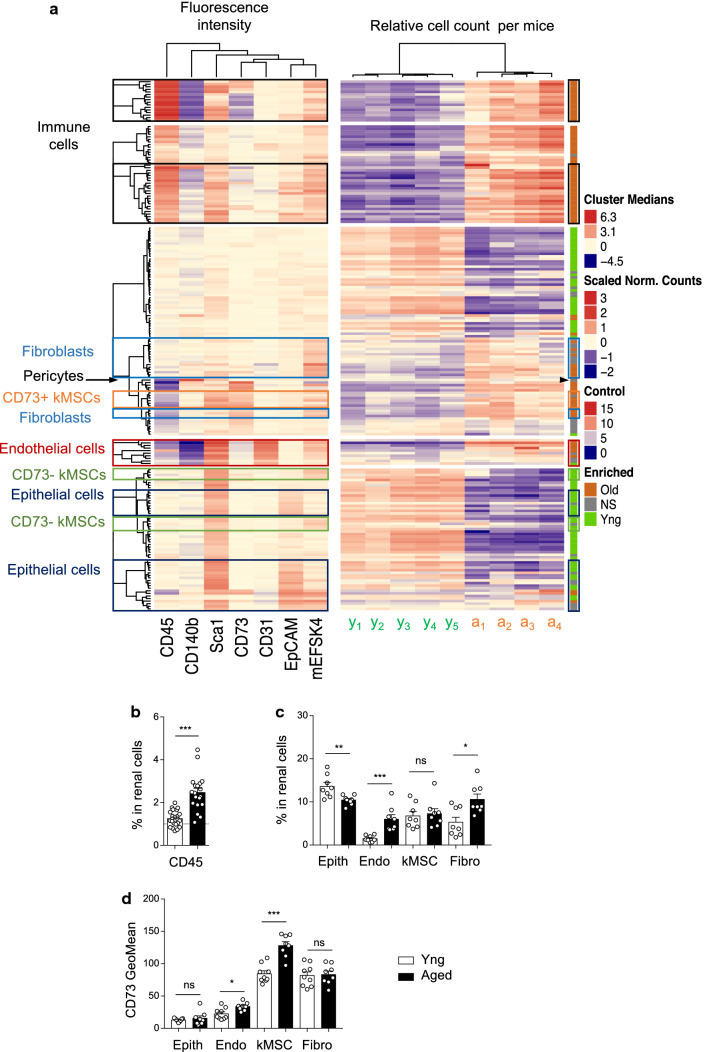
DECyt analysis reveals major changes in renal stromal cell subpopulations with aging. **a** Heatmap representation of renal cell clusters from young and aged mice by DECyt analysis, (left) Median fluorescence intensities of surface markers per clusters of renal cells, (middle) Relative count per cluster of young (y1–y5, *n* = 5 mice) or aged (a1–a4, *n* = 4 mice) renal cells. (Right) Clusters significantly enriched in young (green) or aged (orange) cells, *P* ≤ 0.01 by DESeq2. Not-significant (NS, grey). Hierarchical clustering identified clusters of immune cells (CD45^+^), endothelial cells (Endo, CD31^+^), tubular epithelial cells (Epith, EpCAM^+^), pericytes (CD140b^+^), fibroblasts (Fibro, Sca-1^−^ mEFSK4^+^), mesenchymal stromal cells (kMSCs, Sca-1^ +^ mEFSK4^ +^). **b**, **c** Percentage of (**b**) immune cells (young, *n* = 23; aged *n* = 19) (**c**) Epith, Endo, kMSC and Fibro (young, *n* = 8; aged *n* = 8) in renal cells by flow cytometry. **d** Fluorescence geometric mean of CD73 in the indicated renal cell populations (Epith, Endo, kMSC, Fibro) of young (*n* = 9) or aged (*n* = 8) mice. *P < 0.05 ***P < 0.001 ns, not significant, with Mann–Whitney’s *U* test

To investigate if SASP production is associated with modification of renal stromal cell composition, we set-up a multiparametric labeling strategy by flow cytometry to study Sca-1^+^ stromal cells and used hierarchical clustering by the DECyt method to evaluate the respective enrichment of young or aged cells in each cluster (Supp Fig. 2a). A similar strategy previously helped us to identify distinct age-regulated subsets of mesenchymal stromal cells (MSCs) in the heart, characterized by expression of CD73 or CD90 [15]. Each cell cluster is characterized by marker expression profile of the cells (median fluorescence intensity) and the relative abundance of cells from young or aged mice (relative cell count per mice) in the cluster. Statistical analysis of the relative number of cells per mice per cluster helped to identify clusters significantly enriched in young cells (green) or in aged cells (orange) (Fig. 2a; Supp Fig. 2a, b). In the kidney, lineage marker expression identified clusters of immune cells (CD45^+^), Sca-1^+^ tubular epithelial cells (Sca-1^+^ EpCAM^+^), vascular endothelial cells (Sca-1^+^ CD31^+^), mesenchymal stromal cells (kMSCs; Sca-1^+^, mEFSK4^+^), pericytes (CD140b^+^) and fibroblasts (Sca-1^−^, mEFSK4^+^) (Fig. 2a). Most of the clusters significantly enriched in aged cells identified cell subsets of the renal stroma, whereas clusters significantly enriched in young cells were mostly EpCAM^+^ tubular epithelial cells (thick ascending limb and ductal tubules) or negative for all markers, most likely EpCAM negative epithelial cells (Fig. 2a).

To confirm these results, we performed classical flow cytometry analysis of the data (Supp Fig. 3a), which showed increased frequencies of immune cells (Fig. 2b), endothelial cells and fibroblasts (Fig. 2c) in total renal cells with aging and decreased frequencies of EpCAM + tubular epithelial cells (Fig. 2c). These results were in accordance with the analysis of cell diversity in the aging kidney by single cell RNAseq [16].

In the heatmap, kMSCs partitioned into two kind of clusters based on CD73 expression, with CD73^+^ kMSCs enriched in aged kidney and CD73^−^ kMSCs in young (Fig. 2a). Extraction of the data from the DECyt analysis allowed the visualization of Sca-1 and CD73 co-expression by cells of three CD73^+^ kMSC clusters (c137, c149 and c194) compared to all cells (SuppFig. 2b, c) as well as comparison between young and aged cells in these three clusters (SuppFig. 2d). Classical flow cytometry analysis confirmed that both kMSCs and fibroblasts expressed high levels of CD73 but only kMSCs showed significant up-regulation with aging (Fig. 2d). Study of other surface markers related to the mesenchymal lineage such as the integrin ß1 (CD29) and the integrin Vα (CD51) [17] by young and aged kMSCs showed no modification with aging (Supp Fig. 3b). In contrast to cardiac MSCs [15], kMSCs did not express significant surface levels of CD90, CD34 and CD140a (Supp Fig. 3c, d). The overall analysis of parenchymal and non-parenchymal cell clusters in the kidney revealed deep modifications of renal stromal cell composition with aging.

### Aging increased the frequency of renal macrophages producing inflammatory cytokines at steady state and in response to LPS

**Fig. 3:**
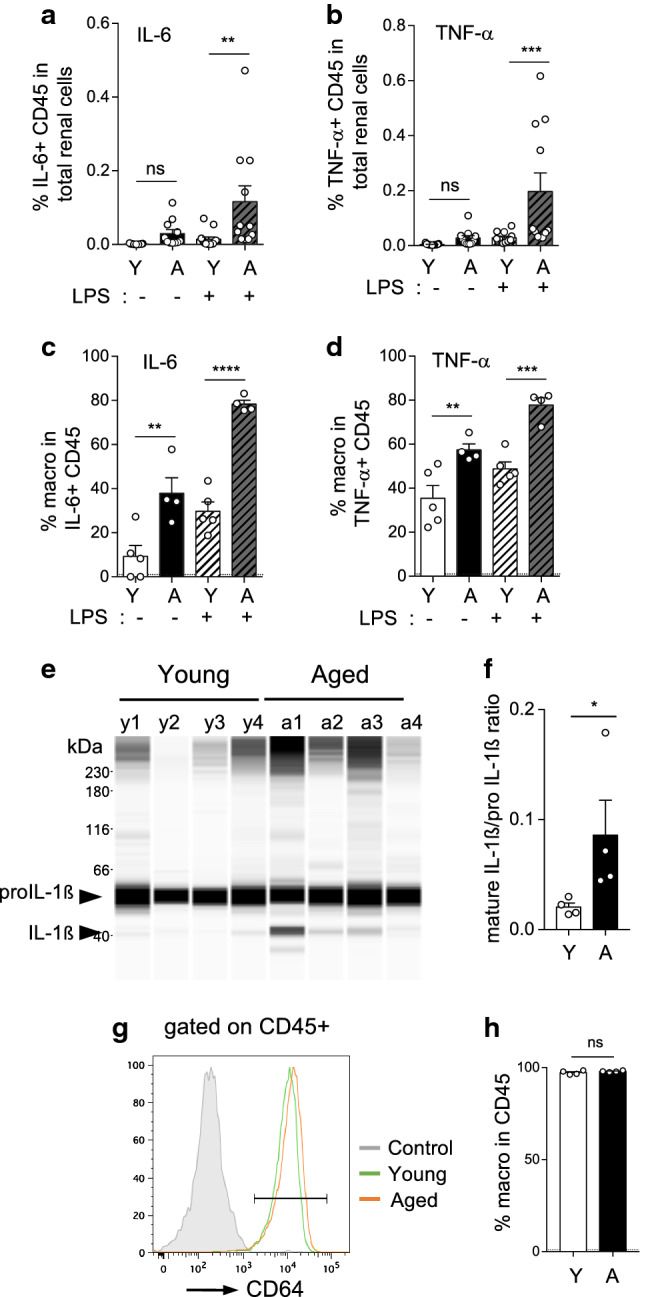
Immune cells are the principal source of pro-inflammatory cytokines in the aging kidney. **a**–**d** Percentage of immune cells (CD45^+^) positive for IL-6 (**a**) or TNF-α (**b**) in total renal cells of young (*n* = 13) or aged (*n* = 11) mice, with or without LPS stimulation for 4 h, determined by flow cytometry after intracellular staining. Percentage of macrophages (CD64^+^ MHCII^+^ CD45^+^) in IL-6^+^ CD45^+^ (**c**) or in TNF-α^+^ CD45^+^ (**d**) are shown (young, *n* = 5; aged *n* = 4). **e**, **f** Capillary western blot image (**e**) and ratio quantification (**f**) of mature IL-1ß and pro-IL-1ß in protein extracts of CD45^+^ immune cells, cell sorted by flow cytometry from primary cell cultures of young and aged kidneys at day 7 (*n* = 4 mice per group). **g**, **h** Flow cytometry analysis of primary cell cultures of young or aged kidneys (*n* = 4 per group) showing the percentage of macrophages (CD64^+^) in CD45^+^ immune cells at day 7. Significant differences were evaluated by Two-way RM ANOVA with Sidak’s post hoc test (**a**–**d**) **P < 0.01, ***P < 0.001, ****P < 0.0001, *P < 0.05, ns, not significant, or with Mann–Whitney *t* test (**f**, **h**). **a** Interaction *P* = 0.0261; Age *P < 0.05; LPS **P < 0.01, **b** Interaction *P *= 0.0163; Age **P < 0.01; LPS **P < 0.01, **c** Interaction *P* = 0.0085; Age ***P < 0.001; LPS ****P < 0.0001, **d** Interaction ns; Age **P < 0.01; LPS **P < 0.01

The DECyt analysis of renal stroma highlighted significant numbers of clusters of CD45^+^ immune cells enriched with aging in the kidney (Fig. 2a), concordant with the recently reported age-related changes in diversity of renal immune cells, based on the analysis of single-cell RNA seq diversity score [16]. Pro-inflammatory cytokine production was evaluated in renal cell suspension from young or aged mice by intracellular cytokine staining just after enzymatic tissue digestion, using LPS stimulation to reveal activation cell state (Fig. 3a–d; Supp Fig. 4a). Cells positive for high IL-6 or TNF-α expression were identified within the CD45^+^ immune cell population, whereas no significant staining was observed for endothelial cells or kMSCs (Supp Fig. 4a). Aging significantly increased the frequency of IL-6^+^ or TNFα^+^ immune cells in total renal cells compared to young after LPS stimulation (Fig. 3a, b). Interestingly, in aged mice, the frequency of macrophages in total renal cells increased (SuppFig. 4b) as well as their activation state. Indeed, aging increased the proportion of macrophages producing IL-6 or TNF-α cytokines in CD45^+^ at steady state and after LPS stimulation (Fig. 3c, d). Immune cells, sorted by flow cytometry from primary renal cell cultures of aged mice, were characterized by increased ratio of mature IL-1ß over pro-IL-1ß protein compared to young (Fig. 3e, f). Interestingly, analysis of the CD45^+^ cells in primary renal cell cultures revealed these cells were mainly macrophages (Fig. 3g, h).

These results clearly demonstrated that immune cells in the renal stroma, in particular macrophages, play a major role in the production of pro-inflammatory mediators during aging.

### Aging increased the frequency of CCR2^+^ macrophages with a pro-inflammatory profile in the kidney

**Fig. 4:**
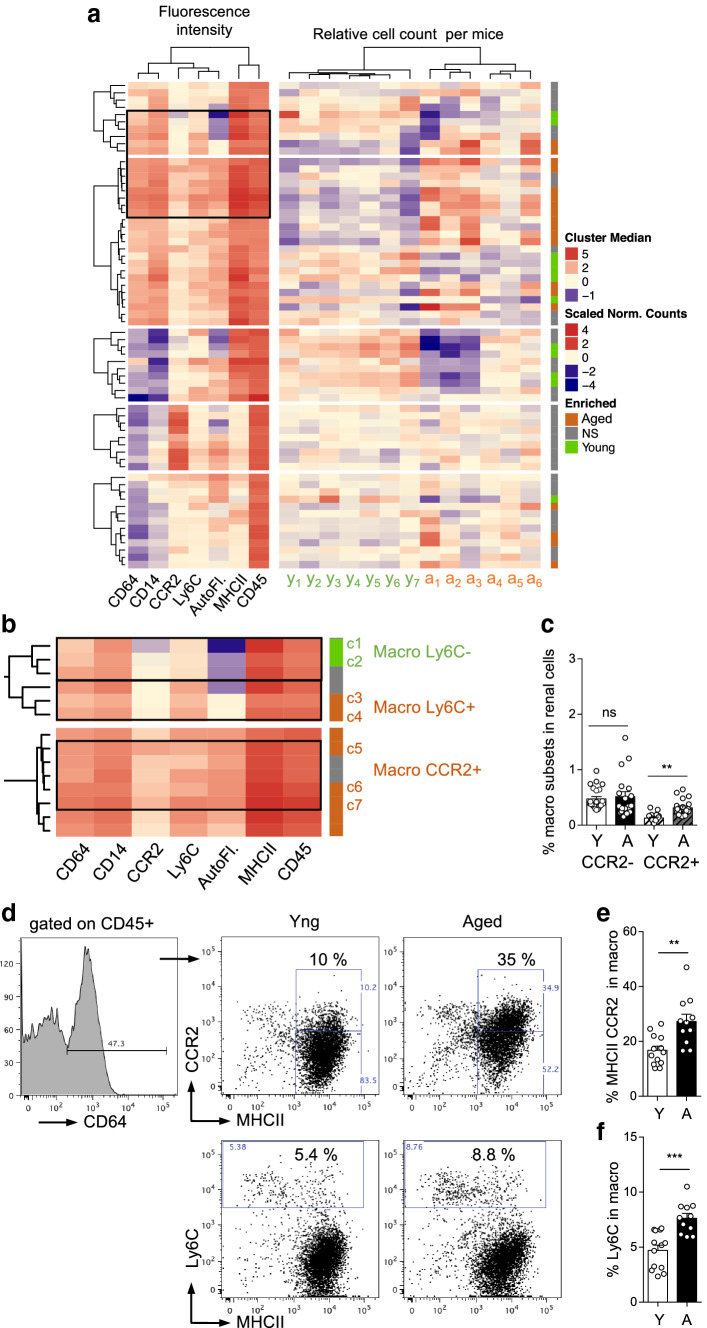
DECyt analysis and phenotypic characterization of renal macrophage subsets modified with aging. **a**, **b** Heatmap representation of CD45^+^ renal immune cell clusters from young and aged mice by DECyt analysis (**a**), (left) Median fluorescence intensities of surface markers and autofluorescence (AutoF.) per clusters of CD45^+^ renal immune cells (middle) Relative count per cluster of young (y1–y7, *n* = 7 mice) or aged (a1–a6, *n* = 6 mice) renal immune cells. (Right) Clusters significantly enriched in young (green) or aged (orange) cells, *P* ≤ 0.01 by DESeq2. Not-significant (NS, grey). **b** Enlargement of some macrophage clusters. **c** Percentage of CCR2^−^ or CCR2^+^ macrophages in renal cells of young (*n* = 23) or aged (*n* = 19) mice (**d**–**f**) Representative dot-plots of MHCII and CCR2 or Ly6C expression by CD64^+^ macrophages (**d**), percentage of CCR2^+^ in CD64^+^ MHCII^high^ macrophages (**e**), percentage of Ly6C in CD64^+^ macrophages (**f**) of young (*n* = 13) or aged (*n* = 11) mice. **P < 0.01 ***P < 0.001, ns, not significant with Mann–Whitney *t* test

Macrophages are well-known contributors to kidney damage resulting from various stresses [8, 18–20], and, thanks to single-cell gene expression analysis, composition of renal macrophage clusters has been shown to be influenced by age [16]. Since, we previously observed increased frequencies of blood CCR2^+^ monocytes and cardiac CCR2^+^ macrophages with aging [15], we performed DECyt analysis of CD45^+^ renal cells from aged mice compared to young to better characterize the age-related macrophage populations of the kidney (Fig. 4a, b). Macrophages were identified in CD45^+^ renal immune cells, based on CD64 expression as previously described [6, 7, 9, 11], and monocyte-derived macrophages were studied based on MHCII, CCR2 and Ly6C expression (Fig. 4a–f).

The DECyt analysis identified several clusters significantly enriched in macrophages from aged mice. Compared to the clusters enriched in young cells (c1, c2), clusters enriched in aged macrophages are characterized by higher expression of Ly6C (c3, c4, c5, c6 and c7) and for some of them, higher expression of CCR2 (c5, c6 and c7) (Fig. 4b). Classical flow cytometry analysis confirmed these results with increased frequency of the CCR2^+^ subset in macrophages and in total renal cells, whereas the frequency of renal CCR2^−^ macrophages in total renal cells was not modified with aging (Fig. 4c–e). In addition, up-regulation of Ly6C expression by macrophages with aging (Fig. 4d, f), strongly suggest that CCR2^+^ macrophages in the aged kidney are related to newly recruited inflammatory monocyte-derived macrophages.

**Fig. 5:**
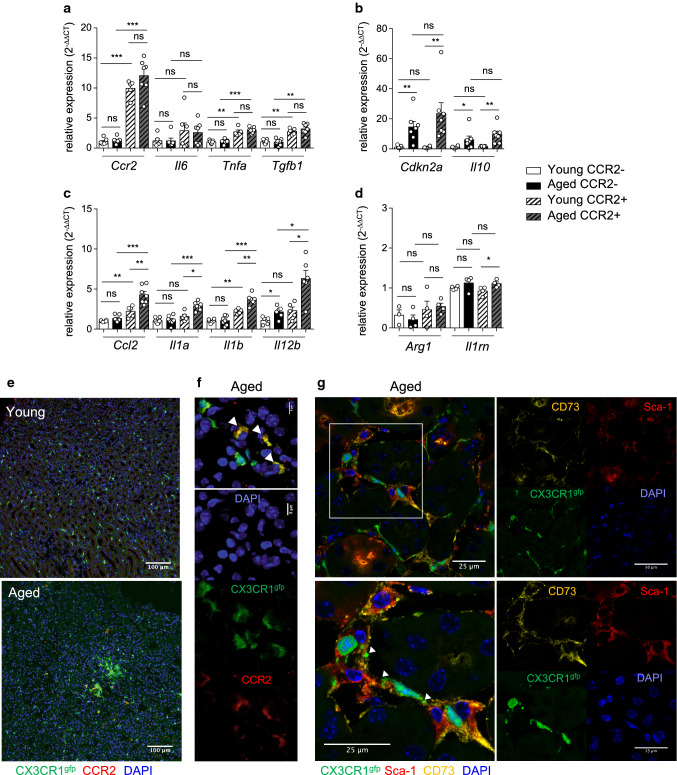
Up-regulation of pro-inflammatory mediator expression by CCR2^+^ macrophages with aging in the kidney. **a**–**d** Gene expression of CCR2^+^ and CCR2^−^ renal macrophage subsets cell sorted from young or aged kidneys. Results are expressed as relative expression compared to young CCR2^−^ macrophages. (*n* = 5–7). **e**–**g** Images by confocal microscopy of macrophages in the kidney of young (**e**) and aged (**f**, **g**) CX3CR1^gfp/+^ mice. Anti-CCR2 (red), DAPI (nuclei), gfp (green) (**e**, **f**). Scale bars are indicated (**e**, 100 µm), (**f**, 8 µm). Arrows showed macrophages co-expressing CCR2 and CX3CR1 (**f**). Co-localization of kMSCs stained with anti-CD73 (yellow) and anti-Sca-1 (red) and of macrophages (gfp, green) in kidneys from aged CX3CR1^gfp/+^ mice with enlarged image (**g**). Nuclei stained with DAPI (blue). Scale bar (25 µm), Arrows indicated macrophage and kMSC interactions. *P < 0.05 **P < 0.01 ***P < 0.001 ns, not significant, with Mann–Whitney’s *U* test

To determine if aging differently impacted gene expression by renal macrophage subsets, macrophages were cell sorted by flow cytometry based on CCR2 expression (Fig. 5a–d). Gene expression of *Tnfa*, *Il6* and *Tgfb1* were not modified with aging, with higher expression of *Tnfa* and *Tgfb1* by the CCR2^+^ macrophage subset compared to the CCR2^−^ macrophage subset (Fig. 5a). In both macrophage subsets, aging-induced gene expression o*f Il10* and of the classical senescence associated Cdki, *Cdkn2a* (Fig. 5b). At the contrary, aging specifically increased mRNA expression of several pro-inflammatory mediators (*Ccl2, Il1a, Il1b, Il12b*) by the CCR2^+^ macrophage subset (Fig. 5c) but did not significantly modify mRNA expression of M2-related markers (*Arg1, Il1rn*) (Fig. 5d). Using confocal microscopy, we could visualize few macrophages, co-expressing CX3CR1 and CCR2, in the medulla of aged kidneys of CX3CR1^gfp/+^ mice, located within immune cell aggregates (Fig. 5e, f).

These results identified the renal monocyte-derived CCR2^+^ macrophage subset as a key player in the production of pro-inflammatory mediators with aging, due to increased frequencies in the kidney as well as increased activation state. Interestingly, confocal microscopy identified some CX3CR1^gfp/+^ macrophages tightly associated with CD73^+^ kMSCs in the interstitial space of the medulla in aged kidneys of CX3CR1^gfp/+^ mice (Fig. 5g) suggesting that crosstalk between these two age-regulated renal stromal cell subsets could modulate their functions.

### Paracrine factors from the renal micro-environment impact the inflammatory response of monocytes

**Fig. 6:**
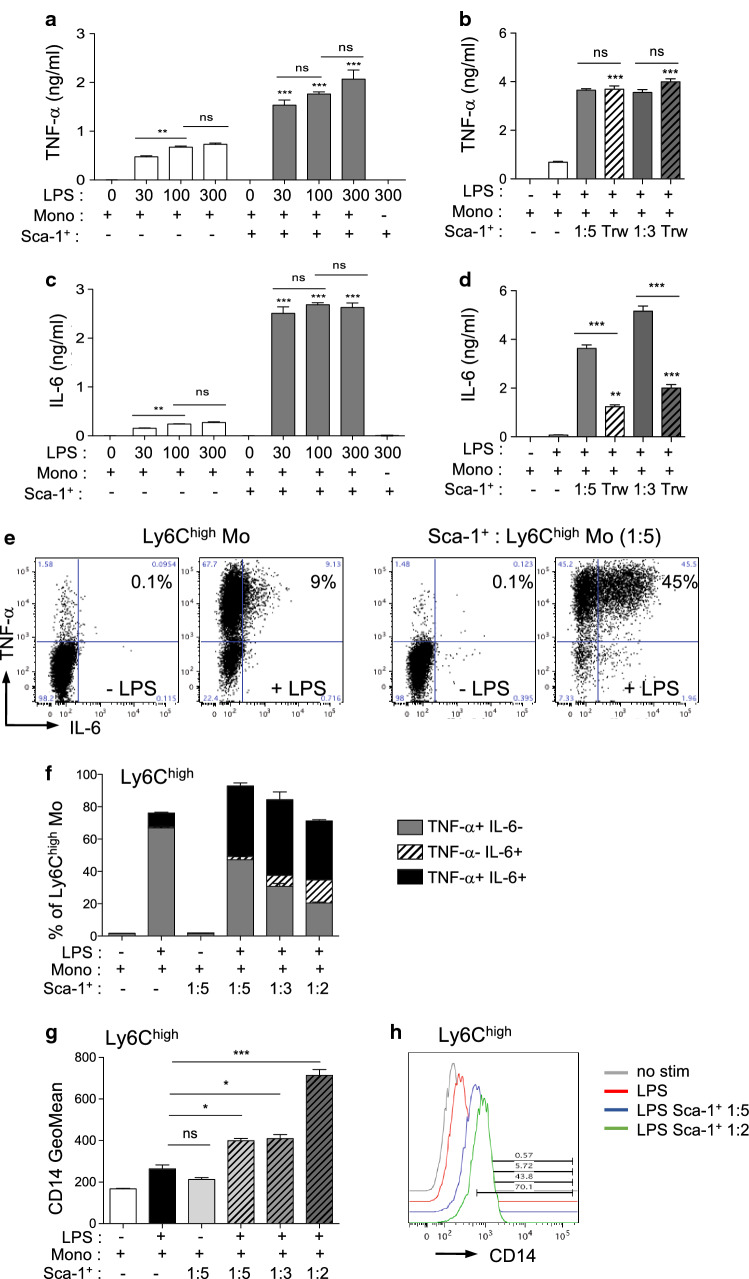
Monocyte/macrophage crosstalk with Sca-1^+^ renal cells modulates inflammatory responses. **a**–**d** TNF-α (**a**, **b**) and IL-6 (**c**, **d**) concentrations in co-culture supernatants of monocytes (mono) and Sca-1^+^ renal cells (Sca-1^+^) stimulated or not for 24 h with LPS (**a**, **c**) Co-cultures (ratio Sca-1^+^ : mono, 1:5) treated with increasing doses of LPS (0–300 ng/ml) and compared to monocytes or Sca-1^+^ alone. **b**, **d** Co-cultures of monocytes and Sca-1^+^ renal cells performed without or with transwells (Trw, Mo in the upper chamber) were stimulated for 24 h with 300 ng/ml LPS (ratio Sca-1 + : mono, 1:5 or 1:3) (*n* = 8). **e**, **f** Intracellular staining of IL-6 and TNF-α in Ly6C^high^ CX3CR1^low^ monocytes co-cultured or not with Sca-1^+^ renal cells and stimulated or not with 300 ng/ml LPS for 4 h. Representative dot plots (**e**) and histograms (**f**) showed percentages of IL-6 + and/or TNF-α  + in monocytes for the different culture conditions (*n* = 8). **g**, **h** Fluorescence geometric mean (**g**) and representative histogram (**h**) of CD14 expressed by Ly6C^high^ CX3CR1^low^ monocytes co-cultured or not with Sca-1^+^ cells at different ratio (ratio Sca-1^+^ : mono, 1:5, 1:3 or 1:2) and stimulated or not with 300 ng/ml LPS for 4 h. **P < 0.01 ***P < 0.001 ns, not significant, unless indicated, groups are compared to LPS treated Mo alone, One-way ANOVA with Bonferroni’s post-test

It has been shown, in several organs, that tissue-derived factors play critical roles in driving the acquisition of effector functions by monocyte-derived macrophages [12, 13]. We focused our analysis on Sca-1^+^ CD31^−^ CD45^−^ cells isolated from the primary renal cell cultures, to test if these cells could modulate the pro-inflammatory cytokine production of monocytes. Monocytes, co-cultured or not with Sca-1^+^ cells, were stimulated with increasing doses of LPS for 24 h and the production of IL-6 and TNF-α cytokines was tested in culture supernatants (Fig. 6a, c). For both cytokines, co-cultures of monocytes with Sca-1^+^ cells significantly increased their secretion in response to LPS (Fig. 6a, c). To characterize the type of interaction between Sca-1^+^ and monocytes modulating cytokine production, co-cultures were performed with transwells (Fig. 6b, d). Direct cell–cell interactions with Sca-1^+^ renal cells were not required to potentiate TNF-α production by monocytes, suggesting that paracrine mediators were sufficient to induce the maximal production of TNF-α by monocytes (Fig. 6b). In contrast, lack of cell-to-cell contact between monocytes and Sca-1^+^ partially reduced the production of IL-6 by monocytes (Fig. 6d), but still superior to stimulated monocytes alone.

Intracellular cytokine staining revealed that co-cultures with Sca-1^+^ cells increased the production of IL-6 and TNF-α in response to LPS stimulation mainly by up-regulating the frequency of double positive IL-6^+^ /TNF-α^+^ Ly6C^high^ monocytes (Fig. 6e, f; Supp Fig. 5a–e). Conversely, co-cultures in absence of LPS did not modify cytokine production by monocytes (Fig. 6a, c, e, f).

As TLR4 responses are dependent on CD14 co-receptor induction [21], we monitored membrane CD14 expression by Ly6C^high^ monocytes in co-culture experiments (Fig. 6g, h). As expected, LPS treatment induced a rapid up-regulation in CD14 expression by Ly6C^high^ monocytes, which was significantly up-regulated by co-cultures with Sca-1^+^ cells (Fig. 6g, h).

These results identified the Sca-1^+^ renal cells as potent regulators of pro-inflammatory cytokine production in the kidney, in response to TLR4 activation, mainly through paracrine derived factors.

### Aged CD73^+^ kMSCs are still able to promote pro-inflammatory responses of monocytes

We confirmed, in primary cell cultures, that aging modify the Sca-1^+^ renal cell subsets with increased frequency of CD73^+^ kMSCs and decreased frequency of EpCAM^+^ Sca-1^+^ tubular epithelial cells (Fig. 6a, b), as observed in vivo (Fig. 2a). The culture helped to select viable kMSCs and allowed us to test their immuno-regulatory functions in vitro.

**Fig. 7:**
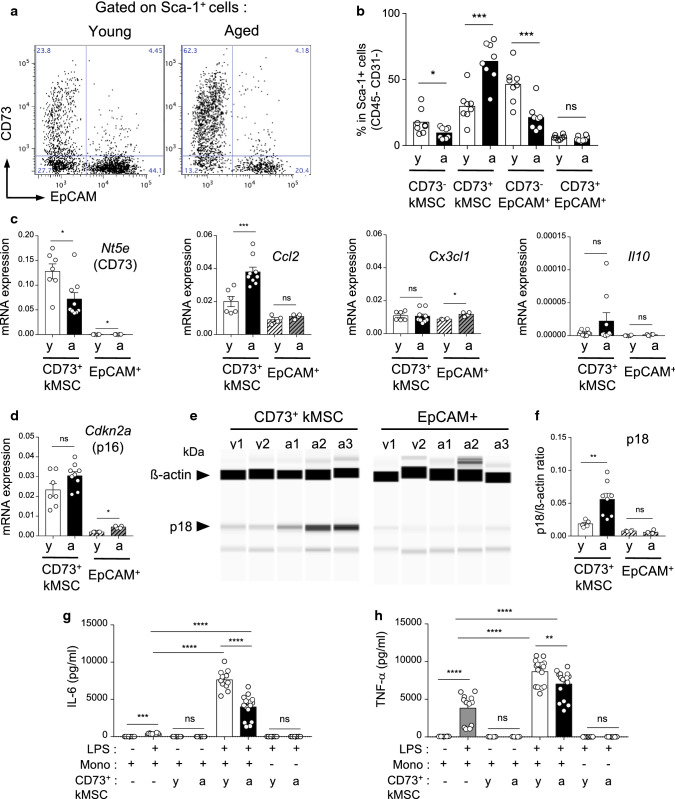
Senescence and immuno-modulatory function of CD73^+^ kMSCs with aging. **a**, **b** Representative dot-plots (**a**) and percentage (**b**) of CD73^+^ kMSCs in Sca-1^+^ renal cells from young or aged kidneys after 7 days of culture (*n* = 8 per group). **c**, **d** Gene expression of *Nt5e* (CD73), *Ccl2*, *Cx3cl1*, *Il10* (**c**) and *Cdkn2a* (p16) (**d**) by young or aged CD73^+^ kMSCs (*n* = 7–9 per group) and epithelial cells (*n* = 4 per group) relative to house-keeping gene. **e**, **f** Capillary western blot image (**e**) and quantification (**f**) of p18 protein expression relative to ß-actin in protein extract of young or aged CD73^+^ kMSCs (*n* = 5–8 per group) and epithelial cells (*n* = 4 per group). (**g**, **h**) IL-6 (**g**) and TNF-α (**h**) concentrations in co-culture supernatants of monocytes (mono) and young or aged CD73^+^ kMSCs stimulated or not for 24 h with LPS (ratio Sca-1^+^ : mono, 1:5) (*n* = 11–15 per conditions). *P < 0.05 **P < 0.01 ***P < 0.001 ns, not significant, with Mann–Whitney *t* test or Kruskal–Wallis with Dunn’s post-test

Aging differently modulated the gene expression of key chemokines involved in monocyte recruitment. As shown in Fig. 7c, aged CD73^+^ kMSCs expressed higher levels of *Ccl2*, whereas aged Sca-1^+^EpCAM^+^ had increased *Cx3cl1* gene expression, while, in both subsets, *Il10* was not significantly modified with aging.

Aged CD73^+^ kMSCs did not up-regulate growth factor and cytokine mRNA classically associated with the acquisition of SASP such as *Il6* or *Igf1* (Supp Fig. 6a, b) nor the classical senescence associated Cdkis, p16^INK4A^ (*Cdkn2a*) (Fig. 7d) and p21^Cip1^ (*Cdnk1a*) (Supp Fig. 6c). At the contrary, mRNA expression of *Cdkn2a* and *Igf1* was increased in aged Sca-1^+^ EpCAM^+^ albeit at low levels (Fig. 7d, Supp Fig. 6b).

Despite the lack of p16 induction, aged CD73^+^ kMSCs had reduce proliferation rate (% Ki67, Supp Fig. 6d, e) and increased γH2AX foci (Supp Fig. 6f, g) suggestive of senescence. As p18^INK4C^ has been shown to be induced in the absence of p16^INK4A^ expression [22], we tested the expression of p18^INK4C^ protein. We observed specific induction of p18^INK4C^ in aged CD73^+^ kMSCs but not in aged Sca-1^+^ EpCAM^+^ suggesting that p18^INK4C^ could constitute an alternative senescence pathway in aged CD73^+^ kMSCs.

We then asked if these age-related changes could modify the immuno-regulatory functions of CD73^+^ kMSCs. We so compared the ability of aged versus young CD73^+^ kMSCs to promote IL-6 and TNF-α cytokine production by monocytes. Aged CD73^+^ kMSCs co-cultured with monocytes, were still able to promote higher IL-6 and TNF-α production in response to LPS compared to LPS-treated monocytes. However, aged CD73^+^ kMSCs induced lower levels pro-inflammatory cytokines compared to young (Fig. 7g, h).

These results showed that specific cell subsets within renal stroma were modified with ageing and could cooperate to promote an inflammatory microenvironment. It will be important to determine which molecular pathways controls this pro-inflammatory cross-talk to decrease incidence of chronic disease in the elderly.

## Discussion

The kidney microenvironment involves a variety of cell populations such as immune cells, endothelial cells or mesenchymal stromal cells (kMSCs) that are known to play an important role in kidney homeostasis and function. The accumulation of senescent cells in the tissues with ageing and SASP production has been demonstrated to play a key role in the progression of organ dysfunction. However, the relative contribution of specific renal cell subsets to SASP-related factor production in the aging kidney has not yet been characterized.

We have shown that the generation of a pro-inflammatory microenvironment in the aging kidney is mainly dependent on modifications of macrophage subset frequencies and activation status. Aging increased the frequency of renal CCR2^+^ macrophages which express higher level of pro-inflammatory cytokine mRNAs such as *Il1b* and *Il12b* compared to young CCR2^+^ macrophages, M1-type cytokines preferentially enriched in monocyte-derived CD11b^high^, F4/80^+^ CD64^+^ macrophages compared to F4/80^high^ CD11b^low^ CD64^+^ macrophages at steady state [9] or in response to injury [20]. These results are in accordance with the enrichment of M1-related genes reported by Almanzar et al*.* for the cell cluster enriched in aged macrophages compared to the cell cluster enriched in young macrophages in the kidney [16]. Interestingly, in the heart, aging also increases the frequency of the monocyte-derived CCR2^+^ macrophage subset within the macrophage pool and is associated with higher IL-1ß cytokine production [15]. As the frequency of blood Ly6C^+^CCR2^+^ inflammatory monocytes is higher in aged mice [15], it is conceivable to propose that the CCR2^+^ macrophage subset in both organs is fueled by increased recruitment of inflammatory monocytes in the tissues through the release of SASP derived CCR2 ligands such as the CCL2 and CCL8 chemokines with aging [23] or in response to tissue injury in the kidney [18, 20] or the heart [24]. Both CCR2^+^ and CCR2^−^ macrophage subsets in the kidney expressed high *Cdkn2a* mRNA levels with aging, but the senescence state of p16 expressing macrophages remains a controversial issue. Up-regulation of *Cdkn2a* expression per se is not sufficient to characterize senescence and it has been shown that p16 expression in macrophages is tightly associated with macrophage activation and polarization [25, 26]. The alteration of cellular functions such as proliferation rate, phagocytosis and the characterization of nuclear abnormalities (decrease expression of lamin B1, increased number of telomere-associated foci) and of lysosomal dysfunction will be helpful to fully address the senescence state of macrophage subpopulations. Grosse et al. have recently shown using a new p16 reporter mice model that treatment with Dasatinib Quercetin combination ablated p16^high^ macrophages in the liver and the abdominal fat of 10-month-old mice [27]. In line, previous study showed that elimination of p16 expressing cells within the splenic environment of p16-3MR mice after ionizing radiation exposure improved immune cell functions such as macrophage phagocytosis [28]. Hence, specific targeting of the pro-inflammatory p16^high^ CCR2^+^ macrophage subset in aged individuals could alleviate side-effects of some senolytic treatments related to the loss of p16^+^ resident pro-regenerative macrophage subsets.

We hypothesized that age-related changes in tissue-derived micro-environmental factors could sustain recruitment and activation of CCR2^+^ monocyte-derived macrophages in the kidney. It would be interesting to further address the heterogeneity of monocyte-derived macrophages in the aged kidney depending on their localization in the tissue (renal cortex versus medulla) and their proximity to CD73^+^ kMSCs by spatial transcriptomic. Aging modified the Sca-1^+^ renal cell subsets with increased frequency of CD73^+^ kMSCs and decreased frequency of EpCAM^+^ Sca-1 ^+^ tubular epithelial cells likely from distal tubule and collecting duct [29], as recently reported, for the ascending limb, thanks to single-cell gene expression signature analysis [16]. The use of EpCAM as an exclusion marker for Sca-1^+^ epithelial tubular cells in the DECyt method did not allow the precise analysis of age-related changes concerning the different tubular segments as more proximal tubules are low to negative for EpCAM [30]. In addition, we observed hybrid-like cell clusters, positive for EpCAM, CD73 and kMSC markers which were most present in aged mice, and required further investigation to rule out possible partial epithelial–mesenchymal transition [31].

In co-cultures with monocytes, Sca-1^+^ renal cells promoted higher percentages of double positive IL-6 and TNF-α monocytes in response to LPS compared to LPS-treated monocytes alone. Monocytes stimulated with aged CD73^+^ kMSCs produced higher pro-inflammatory cytokine in response to LPS compared to monocytes alone, although less efficiently than young CD73^+^ kMSCs, in a per cell basis. In the context of ageing, increased frequency of CD73^+^ kMSC subpopulations could provide additional niche factors to newly recruited monocytes favoring a positive regulatory loop in response to local inflammation. It will be important to identify which are the paracrine factors that control this pro-inflammatory cross-talk. The age-related factors responsible for the increase frequency of CD73^+^ kMSCs in the kidney remained to be determined but IL-1ß treatment in vitro did not up-regulate CD73 expression by kMSCs (not shown).

The 5′ ectonucleotidase CD73 is the rate-limiting enzyme-producing adenosine from extracellular AMP, which is generated from ATP by CD39, an ectonucleoside triphosphate diphosphohydroxylase [32]. In mice, CD73 expression has been shown to be protective against kidney fibrosis and the progression of chronic kidney diseases. Mice deficient for CD73 have reduced adenosine levels and increased pro-inflammatory infiltrate associated with renal fibrosis [33] and exacerbated renal ischemia injury (IRI) [34]. Co-cultures treatment with the CD73 inhibitor AMP-CP did not significantly modified TNF-α and IL-6 production in response to LPS suggesting that paracrine factors derived from CD73^+^ kMSCs, which promote monocyte activation, were independent from CD73 enzymatic activity (not shown).

Our increased understanding of the immuno-plasticity of MSCs has revealed an important role of priming factors, which influence the outcome of MSC immunomodulatory functions [35, 36]. In this context, TLR4 stimulation skews MSCs from diverse tissue sources (bone-marrow, adventitia) toward an MSC1 phenotype, promoting monocyte/macrophage pro-inflammatory responses [35, 37]. The origin of the low chronic inflammatory grade, associated with pathological aging, still remains elusive. However, we can now suspect the involvement of the chronic stimulation of pattern recognition receptors by danger-associated molecular patterns (DAMPs) [38] or pathogen-associated molecular patterns (PAMPs) [39] as a trigger of this inflammation. In older individuals with hypertension, nucleotide metabolites have been associated with inflammasome priming, activation and age-related inflammation; in particular IL-1ß maturation [38].

It remained to determine the molecular determinants driving the cross-talk between macrophages and kMSCs to discriminate between protective pathways involved in the acquisition of tissue-specific functions and microenvironmental factors responsible for the installation of a deleterious inflammatory regulation loop, increasing incidence of chronic diseases in the elderly.

## Methods

### Animals

Male C57Bl/6 mice were purchased from Janvier Laboratories. Young mice (3–5-month-old), and aged mice (20–24-month-old) were used in the experiments as indicated*.* CX3CR1^gfp/+^ CD45.1 C57BL/6 heterozygote reporter mice which express eGFP under control of the endogenous Cx3cr1 promoter and harbor the CD45.1 allele were bred in our animal facilities by backcrossing C57BL/6 CX3CR1^+/+^ CD45.1 males (Jackson laboratory) with C57BL/6 CD45.1 female mice (Charles River). All mice were maintained under specific pathogen-free conditions and handled according to procedures performed in accordance with the recommendations of the European Accreditation of Laboratory Animal Care (86/609/EEC) and guidelines established by the local Ethics and Animal Safety Committee.

### Enzymatic kidney dissociation and cell suspension preparation

Kidney were isolated, released from the renal capsula, minced by scalpel and digested with Liberase TM (Roche) for flow cytometry analysis or with Collagenase Type 2 (Worthington) for kidney stromal cell cultures. Homogenized kidneys were incubated at 37 °C with stirring for 2 × 10 min with Liberase (0.65 WU/ml) in RPMI1640 (Invitrogen) or for 2 × 20 min with Collagenase type 2 (400 UI/ml) in RPMI1640 10% heat inactivated fetal calf serum (FCS, PAA). Cell suspensions were obtained by successive filtration on 100 µm and 40 µm cell strainer filters (BD Falcon) after hypotonic lysis of red blood cells by ammonium chloride (0.83%).

Bone-marrow cells were collected by flushing the femurs and tibia of CX3CR1^gfp/+^ CD45.1 C57BL/6 mice with 2 ml FACS buffer (DPBS, 4% FCS, 2 mM EDTA). Cell suspensions were filtrated on 100 µm cell strainer filters after lysis of red blood cells, centrifuged and counted for further analysis. Monocytes were enriched by negative selection from bone-marrow cell suspensions using EasySep^™^ Monocyte enrichment kit (Stemcell Technologies).

### Flow cytometry and cell sorting

For surface markers, cells were first incubated for 15 min in FACS buffer supplemented with 3% mouse serum, 3% rat serum and 5 µg/ml anti CD16/CD32 (93, Biolegend) to block Fcγ III/II receptors, then incubated for 30 min on ice with conjugated monoclonal antibodies diluted at the optimal concentration in FACS buffer.

Kidney macrophages or monocytes were phenotyped by staining with Pacific Blue anti-Ly6C (HK1.4), APC anti-CD115 (AFS98), APC Cy7 anti-CD45.1 (A20) or APC Cy7 anti-CD45 (30-F11), PE or APC anti-CD64 (X54-5/7.1), PE anti-CD11b (M1/70.15), Pacific Blue or FITC anti I-A/I-E (M5/114.15.2) from Biolegend, PerCP/Cy5.5 anti CD14 (Sa-2-8) from eBioscience and PE anti CCR2 (FAB5538) from R&D Systems.

For intracellular cytokine staining, cells were fixed after surface staining and processed for intracellular staining with PE anti-IL-6 (MP5-20F3) and APC anti-TNF-α (MP6-XT22) or matching isotype controls from ebioscience with the IntraStain kit (Dako).

The anti-mouse antibodies used to phenotype and to cell sort Sca-1 + renal cell populations were purchased from Biolegend or eBioscience: Pacific Blue or Brillant Violet 711 anti-sca-1 (LyA/E, D7), FITC anti-CD29 (HMß1-1), FITC anti-CD146 (ME-9F1), PE anti-CD105 (MJ7/18), PE anti-CD51 (RMV-7), PerCP/Cy5.5 anti-CD31 (390), APC anti-CD73 (TY/11.8), APC anti-CD34 (RAM34), APC Cy7 anti-CD45 (30-F11), PE Cy7 anti CD90.2 (53–2.1), PE anti-CD140a (APA5) and APC anti-CD140b (APB5). Epithelial cells were stained with FITC anti EpCAM (G8.8) or gated off in the dump channel using biotinylated anti-EpCAM and Brillant Violet 570 conjugated streptavidin (Biolegend). Necrotic cells were identified by staining with Live/dead Yellow (analyzer) or Aqua (cell sorter) fluorescent reactive dyes (Invitrogen) accordingly to the manufacturer’s instruction.

Multiparameter acquisition of stained cells were performed on a BD LSRFortessa^™^ (4-lasers, BD Biosciences) and analyzed with FlowJo software (Tree Star).

Kidney macrophages negative for Live/dead Aqua staining were isolated from single cell suspension of enzymatic digested kidneys based on CD45, CD64, MHCII and CCR2 expression. Renal CD45^+^ , CD73^+^ kMSCs (Sca-1^+^ CD73^+^ EpCAM^−^ CD31^−^ CD45^−^) and Sca-1^+^ epithelial cells (Sca-1^+^ EpCAM^+^ CD31^−^ CD45^−^) negative for Live/dead Aqua staining were cell sorted from primary cell cultures based on Sca-1, EpCAM, CD73, CD31 and CD45 expression. High-speed sorting was performed with BD InfluxTM cell sorter (BD Biosciences).

### DECyt method

DECyt method was designed to identify statistically significant differential cell subpopulations by hierarchical clustering flow cytometry data of samples from young and aged individuals as previously described [15]. Briefly, each cluster is characterized by fluorescence median intensity of stained surface markers and by the relative number of cells per individual sample within the cluster. Clusters significantly enriched in young or aged cells were identified using DESeq2 [40]. Heatmaps were generated with the ComplexHeatmap [41] package. The heatmap on the left shows fluorescence median intensity of surface markers for each cluster and the dendrogram shows the relationship between clusters. The heatmap in the middle (Scaled Norm. Counts) shows the relative cell counts per sample within each cluster. Significance, *P* value ≤ 0.01, is shown as a single column heatmap on the right, with color code used to identify clusters enriched in aged cells (orange) or in young cells (green); grey is used for non-significant clusters. Antibody panels used for the DECyt analysis are listed in Supplementary Table S1. Complete list of the clusters for renal stroma and immune cells analysis and their respective parameters are provided in supplementary Table S3 and supplementary Table S4.

### Primary cell cultures

After enzymatic digestion of the kidney, cells were seeded in complete medium consisting of α-MEM w/o ribonucleosides w/o nucleosides with glutamax (Invitrogen) supplemented with 10% heat-inactivated FCS (PAA) and 1% Penicillin, Streptomycin (Sigma-Aldrich).

For primary renal cell cultures, cell suspensions were seeded in one T75 cm^2^ flask (Falcon) per mouse and washed twice the next day with DPBS. Adherent cells were grown in complete medium until sub-confluence (7 days) at 37 °C under 5% CO_2_ in a humidified atmosphere. Cells were trypsinized, counted in trypan blue (Gibco) and phenotyped by flow cytometry. Isolation of Sca-1^+^ renal cells was performed at the first passage by Sca-1 positive selection using magnetic beads (EasySep^™^ Mouse Sca-1 Positive Selection Kit, Stemcell technologies). CD73^+^ kMSCs (CD73^+^ sca-1^+^ , EpCAM^−^, CD45^−^, CD31^−^) and Sca-1^+^ epithelial cells (sca-1^+^, EpCAM^+^, CD45^−^, CD31^−^) were selected from the cultures by high-speed sorting (INFLUX, BD Biosciences). For experiments, each replicate corresponds to cells isolated from individual mice or cells pooled from two to three mice after cell subset selection. Biological tests were performed just after selection or after one passage.

### LPS treatment and co-cultures

For intracellular cytokine detection, cell suspensions obtained by enzymatic digestion of the kidney, monocytes only or monocytes with Sca-1^+^ renal cells were incubated in X-VIVO^™^ 15 serum-free medium (Lonza) in ultralow-attachment 24-well plates (Costar) for 4 h, with or without 300 ng/ml LPS from *E coli* (0111:B4; Sigma-Aldrich) and with Brefeldin-A (10 µg/ml) for the last 2 h.

For quantification of cytokines in medium supernatants of monocytes co-cultures, Sca-1^+^ renal cells or CD73^+^ kMSCs were plated the day before in complete medium at 10 000 (1:5) or 16 666 (1:3) cell/well in 96-well plates or at 30 000 (1:5), 50 000 (1:3) or 75 000 (1:2) cell/well in 24-well plates. The next day, monocytes were seeded at 50 000 cell/well in 96-well plates or 150 000 cell/well in 24-well ultra-low attachment plates (Costar) with Sca-1^+^ renal cells or CD73^+^ kMSCs in X-VIVO^™^ 15 serum-free medium (Lonza) and stimulated with the indicated doses of LPS for 4 h (300 ng/ml) or 24 h (30–300 ng/ml). Co-cultures using transwell devices were performed with monocytes in the upper chamber (0.4 µm cell culture inserts; BD falcon) and Sca-1^+^ renal cells in the lower chamber (24-well companion plates; BD Falcon).

To analyze mRNA expression by adherent cell fraction, cell suspensions obtained by enzymatic digestion of the kidney cells were stimulated or not for 3 h with LPS (300 ng/ml) in 24-well plates (BD Falcon) in complete medium at 37 °C under 5% CO_2_ in a humidified atmosphere then washed gently with DPBS (Sigma-Aldrich) before cell lysis.

### Immunofluorescence and confocal microscopy

Kidneys of 5-month and 20-month CX3CR1^gfp/+^ mice were fixed 6 h in DPBS 3.4% PFA, incubated overnight at 4 °C with 30% sucrose (Sigma-Aldrich) in DPBS and embedded in Tissue-Tek OCT compound (Electron Microscopy Sciences) for cryosection. Tissue slides (10 µm) were incubated twice for 10 min with 100 mM glycine (Sigma-Aldrich) then with blocking buffer [75 mM NaCl (Sigma-Aldrich), 15 mM Na_3_ Citrate (Sigma-Aldrich), 1% BSA (Sigma-Aldrich), 2% rabbit serum (Sigma-Aldrich) and 0.05% saponin (Sigma-Aldrich)] for 1 h at RT. After one wash for 10 min at RT with washing buffer (75 mM NaCl, 15 mM Na_3_ Citrate, 0.05% Tween 20), slides were incubated with primary antibodies, goat anti CCR2 (Santa-Cruz, SC6228) or rat anti Sca-1 (Biolegend, clone E13-161.7) and sheep anti CD73 (AF4488, Novus Biological) diluted in blocking buffer overnight at 4 °C. After washing twice for 10 min in washing buffer, slides were stained for 1 h RT with respective conjugated secondary antibodies, AF594 donkey anti goat (Invitrogen, A11058), AF594 rabbit anti rat (Invitrogen, A21211) or AF647 donkey anti sheep (Invitrogen, A21448) diluted in blocking buffer. After two washes in washing buffer, nuclei were counterstained with 0.5 µg/ml DAPI for 30 min and tissue slides were mounted in fluorescence mounting medium (Dako). Images were acquired with an LSM 780 confocal microscope (Zeiss) and analyzed with Fiji software.

CD73^+^ kMSCs were seeded in 8-well culture chamber slides (Lab-Tek II, Nunc) at 12 000 cells/well and fixed in 3.4% PFA (Sigma-Aldrich), 4% sucrose (Sigma-Aldrich) in DPBS. Cells were incubated for 30 min in blocking buffer (5% Goat serum, 0.2% Triton X100 in PBS) and stained overnight at 4 °C with rabbit anti-γH2AX (Millipore) or rabbit anti-Ki67 (SP6, Cell Marque) as described previously [15]. After three washes with 0.2% Tween20 in PBS, cells were stained for 1 h at RT with AF594 goat anti-rabbit (Invitrogen). Nuclei were stained with DAPI (Invitrogen) and slides mounted with fluoromount medium (Dako). Images were acquired with an LSM 780 confocal microscope (Zeiss). Four to five fields per well were analyzed with Fiji software to calculate the percentage of positive fluorescent cells per nuclei.

### Protein quantification by ELISA and capillary-based western blot

IL-6 and TNF-α concentrations were quantified in 24 h culture supernatants by ELISA (eBioscience) following manufacturer’s instructions and analyzed on a multimode spectra microplate reader (Infinite 200, Tecan) with the Magellan^™^ software (Tecan).

Capillary-based “Simple Western System” (WES, ProteinSimple) was used according to the manufacturer’s protocol to quantify IL1-ß protein, using goat anti mouse IL-1b/ IL-1F2 (AF-401-NA, R&D systems, 1/250), and p18 protein, using rabbit monoclonal anti-p18 (ab192239, Abcam). Protein expression was normalized by ß-actin quantification using rabbit anti-mouse ß-actin (4970, CTS, 1/250) as described previously [15]. Briefly, protein extracts from 50000 sorted cells were adjusted to 0.15 mg/ml concentration. Separation and immunoprobing were performed automatically and the chemiluminescent signals were detected and analyzed by Compass software (ProteinSimple).

### RNA extraction, reverse transcription and real-time PCR

Total RNA was extracted from kidneys using the Qiagen RNeasy plus mini kit (QIAGEN) and quantified by spectrophotometry (ND-100 NanoDrop, Thermofisher). Reverse transcription of mRNA from kidney was performed with the MultiScribe^™^ reverse transcriptase (High-Capacity cDNA Reverse Transcription kit, Applied Biosystem). For cell subsets selected by cell-sorting, ReliaPrep^™^ RNA Miniprep Systems (Promega) and SuperScript^™^ VILO^™^ cDNA Synthesis Kit (Invitrogen) were used as previously described [15]. RT PCR was performed on retro-transcribed RNA with SYBR green (Takara) on ABI StepOne + (Applied Biosystems) or Viia7 (Thermo). Primer sequences are listed in supplementary Table S2. Expression of gene transcripts was normalized to *Rplp0* (36B4) house-keeping gene (2^−∆CT^) or compared to control group (young) with the comparative cycle threshold (CT) method (2^−∆∆CT^).

### Statistical analysis

Results are expressed as means ± SEM. The statistical significance between young and aged groups was estimated with the unpaired student’s two-tailed *t* test or, when indicated in figure legends, with the nonparametric Mann–Whitney’s *U* test. Multiple group comparisons were performed when indicated with two-way repeated measures (RM) ANOVA and Sidak’s post hoc test, by one-way ANOVA with Bonferroni’s post hoc test or for samples with non-gaussian distribution with the Kruskal–Wallis test with Dunn’s post hoc test. Differences between groups were tested with Prism software (version 7, GraphPad Software) and considered statistically significant for *P* < 0.05.

### Supplementary Information

Below is the link to the electronic supplementary material.

Supplementary file1: TableS1 and TableS2 (PDF 5891 KB)

Supplementary file2: TableS3 (XLSX 92 KB)

Supplementary file3: TableS4 (XLSX 65 KB)

Supplementary file4: Supplementary figures (PDF 45 KB)
