# Supplementary material for: Kidney inflammaging is promoted by CCR2+ macrophages and tissue-derived micro-environmental factors
Source: Cell Mol Life Sci. 2020 Dec 12;78(7):3485–501. doi: 10.1007/s00018-020-03719-0 (PMC8038964; doi:10.1007/s00018-020-03719-0)
Supplement: Supplementary file 1 — Supplementary file1: TableS1 and TableS2 (PDF 5891 KB) [file 18_2020_3719_MOESM1_ESM.pdf]

Supplementary Figures

Kidney inflammaging is promoted by CCR2+ macrophages and tissue-derived micro-environmental factors

Lefèvre L, Iacovoni JS, Martini H, Bellière J, Maggiorani D, Dutaur M, Marsal DJ, Decaunes P, Pizzinat N, Mialet-Perez J, Cussac D, Parini A and V Douin-Echinard.

Cellular and Molecular Life Sciences

Corresponding authors :
Pr Angelo Parini
angelo.parini@inserm.fr

Dr Victorine Douin-Echinard
victorine.douin@inserm.fr

Institute of Metabolic and Cardiovascular Diseases, UMR1048, 31432 Toulouse, Cedex 4, France
Paul Sabatier University, 31062 Toulouse, Cedex 9, France

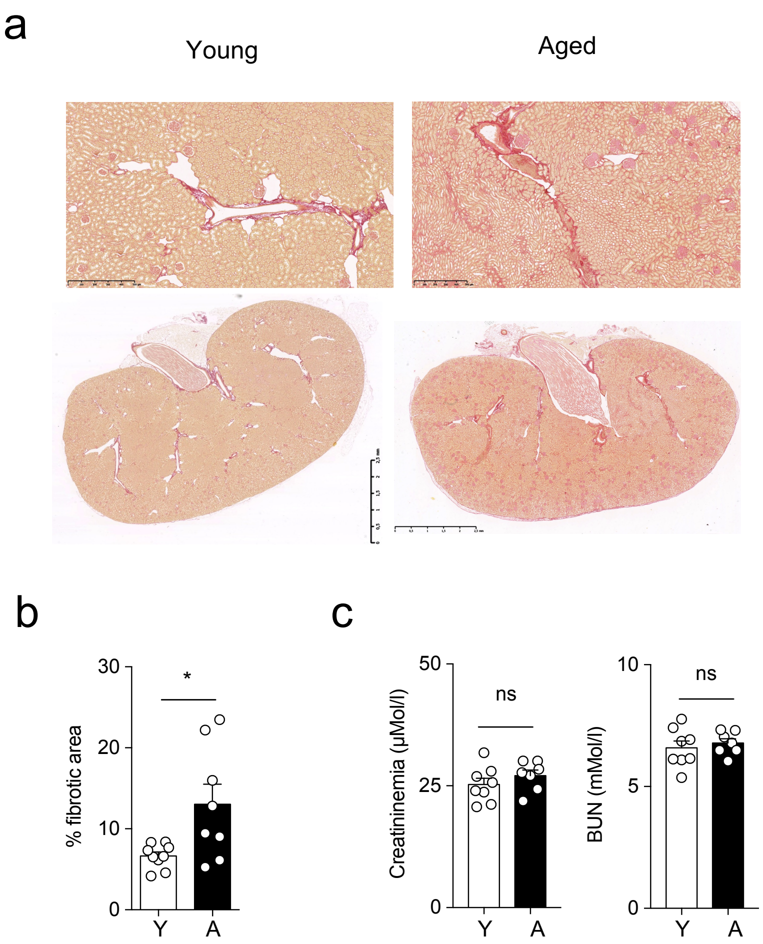

Supplementary Fig. 1 Physiological kidney aging results in mild fibrosis. a, **b** Representative images of young and aged kidneys stained with Sirius Red (**a**) and quantification (**b**) of renal fibrosis (n=8-9 mice per group). **c** Plasma creatinine (left panel) and BUN (right panel) of young and aged mice (n=7-8 mice per group). *P<0.05 ***P<0.001, ****P<0.0001, ns, not significant, Mann Whitney t test.

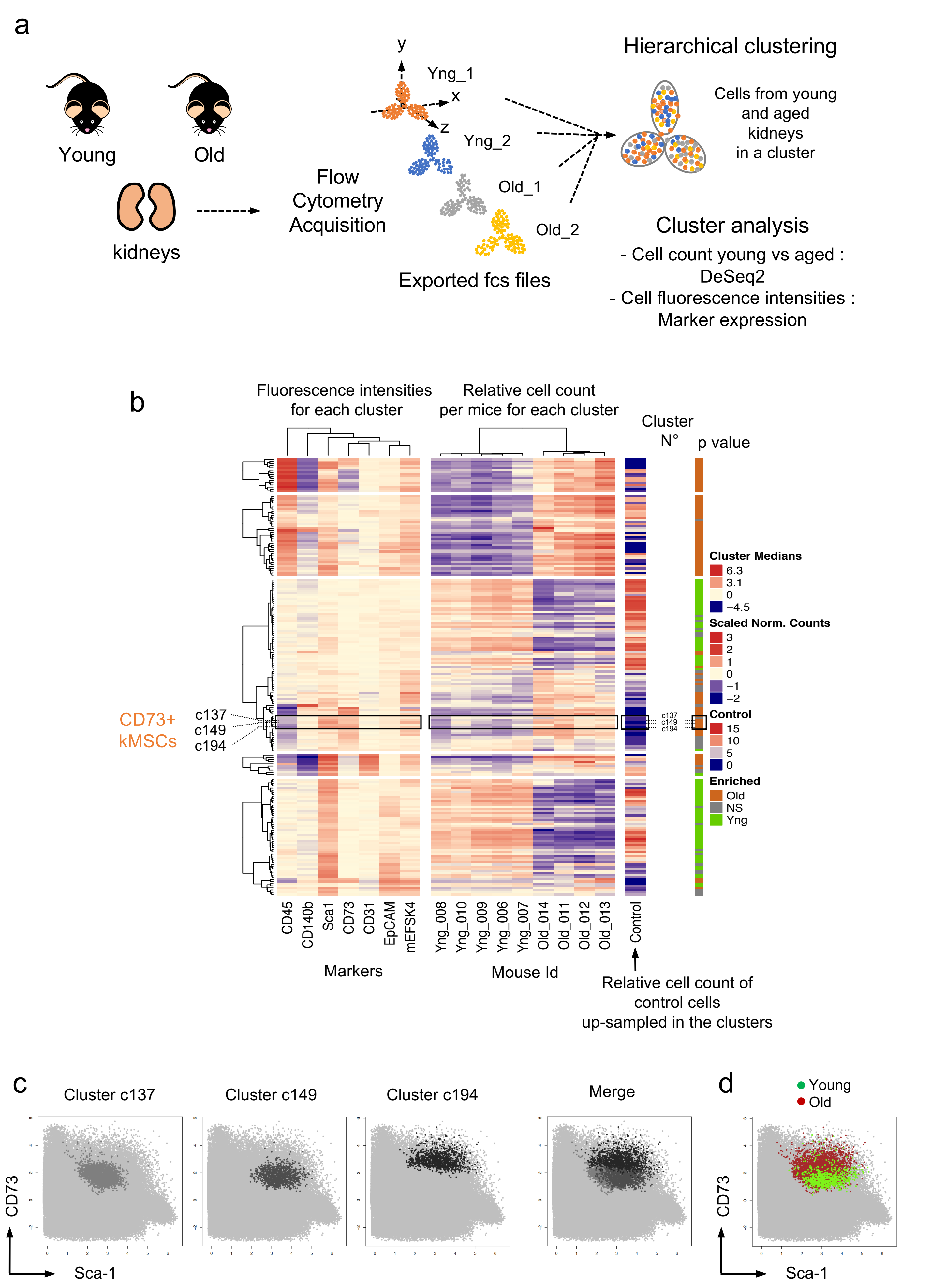
a
Young
Old
kidneys
Flow
Cytometry
Acquisition
y
x
z
Yng_1
Yng_2
Old_1
Old_2
Exported fcs files
Hierarchical clustering
Cells from young
and aged
kidneys
in a cluster
Cluster analysis
- Cell count young vs aged :
DeSeq2
- Cell fluorescence intensities :
Marker expression
b
Fluorescence intensities
for each cluster
Relative cell count
per mice for each cluster
Cluster
N°
p value
CD73+
kMSCs
c137
c149
c194
Cluster Medians
6.3
3.1
0
−4.5
Scaled Norm. Counts
3
2
1
0
−1
−2
Control
15
10
5
0
Enriched
Old
NS
Yng
CD45
CD140b
Sca1
CD73
CD31
EpCAM
mEFSK4
Yng_008
Yng_010
Yng_009
Yng_006
Yng_007
Old_014
Old_011
Old_012
Old_013
Control
Markers
Mouse Id
Relative cell count of
control cells
up-sampled in the clusters
c
Cluster c137
Cluster c149
Cluster c194
Merge
CD73
Sca-1
d
Young
Old
CD73
Sca-1

Supplementary Fig. 2 Examples of CD73+ kMSC clusters identified with the DECyt method. a Flowchart of the DECyt method used to identify aged-related cell clusters in the kidney. Kidneys from young (n=5) and aged (n=4) mice were enzymatically dissociated and processed for flow cytometry by staining with anti Sca-1, anti CD73, anti mEFSK4, anti CD45, anti CD31, anti EpCAM mAbs and with dead cell stain kit to identify viable cells as described in *Material and Methods*. Cells from individual mice were acquired on BD LSR Fortessa. The exported fcs files were used for bioinformatic analysis performing hierarchical clustering of both young and aged cells and defining clusters based on median fluorescence intensity of the surface markers. Statistical analysis by DeSeq2 was performed using the count of the number of cells per cluster per sample to identify significant clusters differentially represented by young versus aged mice (Supplementary Table S3). **b** Heatmap representation of 196 renal cell clusters (rows) from young and aged mice by DECyt analysis showing: Median surface marker fluorescence intensities per cluster (first set of columns, left); Normalized count per cluster (second set of columns, middle) of young (Yng, n=5 mice) or aged (Old, n=4 mice) samples; Cluster number indicates the three clusters used for subsequent dot-plot representation; Up-sampled cluster counts for control cells (Control, right column) stained with Sca-1, CD45 and CD31 mAbs and with isotype controls instead of CD73, mEFSK4, EpCAM and CD140b mAbs and up-sampled in the existing clusters, Cluster significance based on p-values from DESeq2 (rightmost column with green indicating up in young, orange for clusters up in aged and grey for non-signifigant, $P \leq 0.01$). Three CD73+ kMSC clusters (c137, c139, c194) are examples of clusters up in aged with no up-sampling of control sample data (dark blue in the Control heatmap). **c, d** Dot-plots showing Sca-1 and CD73 expression for (**c**) the three selected CD73+ kMSC clusters (grey) compared to all cells (light grey), (**d**) the cells from young (green) or aged (red) kidneys in the three selected CD73+ kMSC clusters. Units are ArcSine transformed fluorescence intensities.

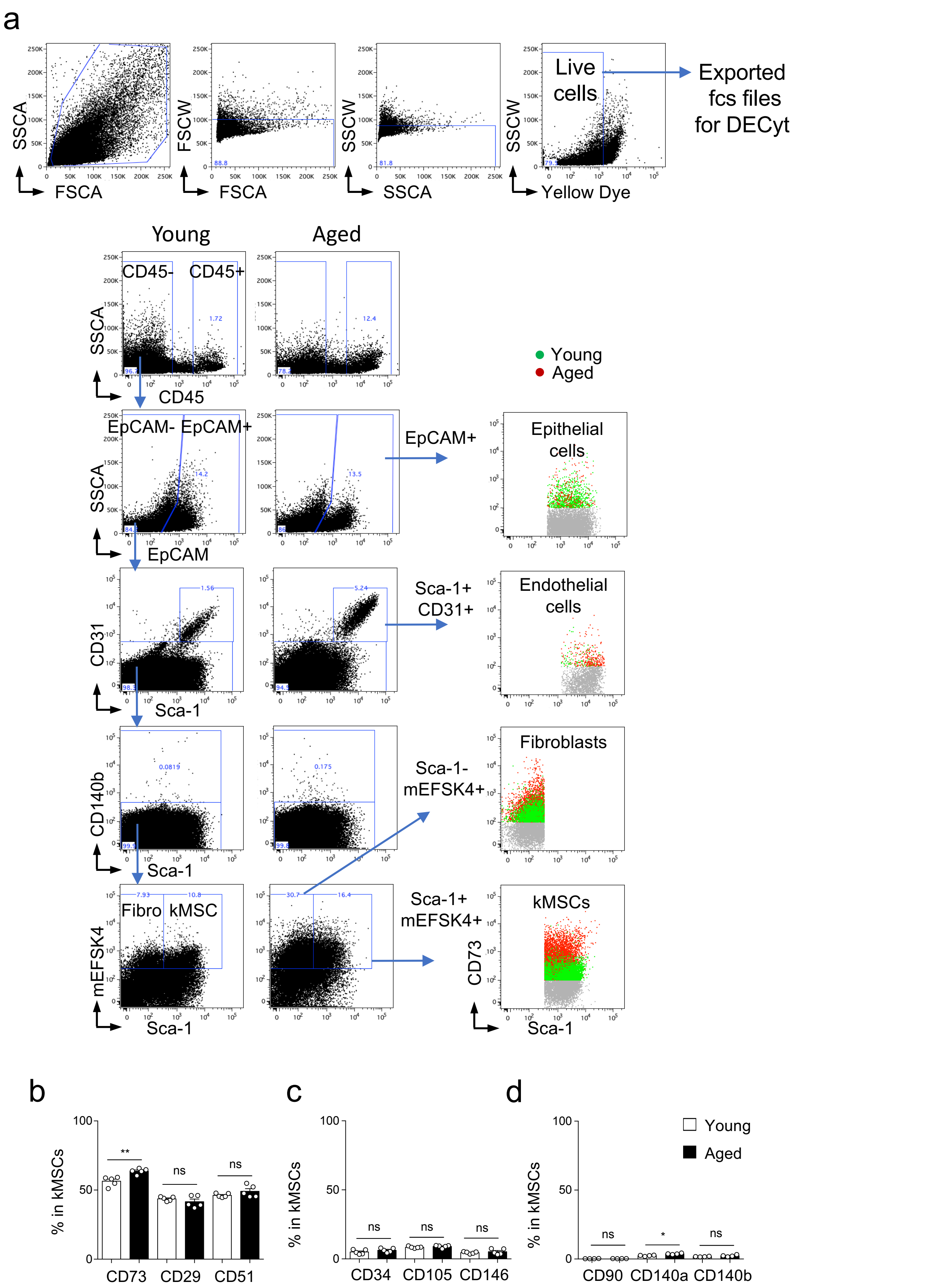
a
SSCA
FSCA
FSCW
SSCW
Yellow Dye
Live cells
Exported fcs files for DECyt
Young
Aged
CD45- CD45+
CD45
EpCAM- EpCAM+
EpCAM
EpCAM+
Epithelial cells
CD31
Sca-1
Sca-1+ CD31+
Endothelial cells
CD140b
Sca-1- mEFSK4+
Fibroblasts
mEFSK4
Fibro kMSC
Sca-1+ mEFSK4+
kMSCs
CD73
b
% in kMSCs
CD73 CD29 CD51
c
CD34 CD105 CD146
d
CD90 CD140a CD140b
Young
Aged

Supplementary Fig. 3 Gating strategy to identify renal cell population by flow cytometry and phenotypic characterization of young and aged kMSCs.

a Representative gating strategy used for classical flow cytometry analysis to validate the identity of renal cell clusters obtained by the DECyt method; immune cells (CD45+), vascular endothelial cells (Sca-1+ CD31+ CD45-), tubular epithelial cells (Sca-1+, EpCAM+), fibroblasts (Sca-1- mEFSK4+ CD31- CD45-) and mesenchymal stromal cells (kMSCs, Sca-1+ mEFSK4+ CD31- CD45-) from young or aged mice were shown. Dot-plots showed young (green) or aged (red) cells positive for CD73 and Sca-1 in each subset. **b**-**d** Percentage of mesenchymal-related markers (CD73, CD29, CD51) (**b**), endothelial related markers (CD34, CD105, CD146) (**c**) and CD90, CD140a, and CD140b (**d**) in kMSCs by flow cytometry. Immune cells (CD45+), endothelial cells (CD31+) and epithelial cells (EpCAM+) were excluded from the analysis. $^*P<0.05$ $^{**}P<0.01$ ns, not significant, with Mann Whitney t test.

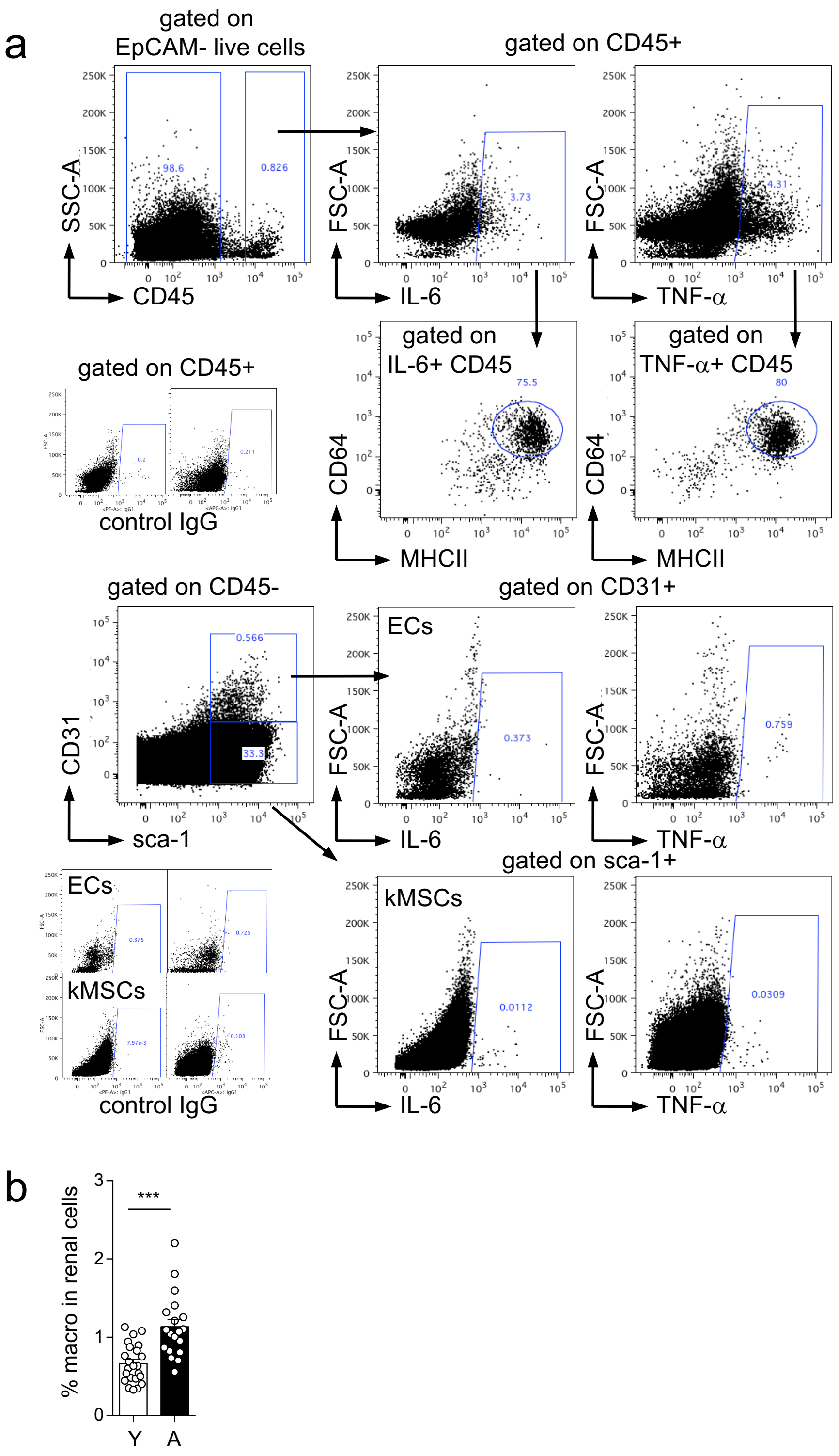

Supplementary Fig. 4 Gating strategy to identify IL-6+ or TNF-α+ renal stromal cells by flow cytometry. a Representative gating strategy used to identify the IL-6+ and TNF-α+ positive cells in young and aged kidneys determined by flow cytometry after intracellular cytokine staining. Total immune cells (EpCAM-, CD45+), macrophages (EpCAM-, CD45+, CD64+, MHCII+), endothelial cells (ECs : EpCAM-, CD45-, CD31+, Sca-1+), kidney mesenchymal stromal cells (kMSCs : EpCAM-, CD45-, CD31-, Sca-1+). Intracellular staining with isotype controls are shown for CD45, ECs and kMSCs. **b** Percentage of macrophages (CD45+, CD64+, MHCII+) in renal cells of young (n=23) or aged (n=19) mice by flow cytometry. ***P<0.001, Mann Whitney t test.

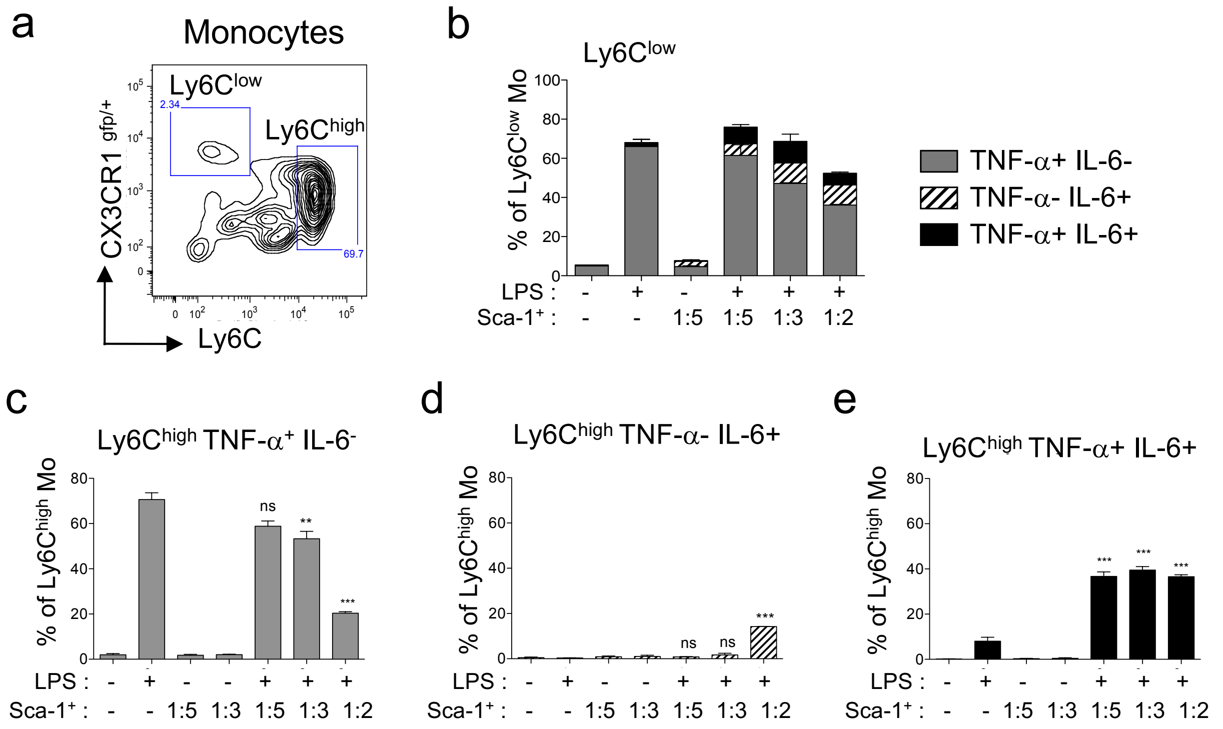

Supplementary Fig. 5 Co-culture with Sca-1+ renal cells regulates monocyte response to inflammatory stimulus.

a Representative dot plot showed monocyte subsets based on Ly6C and CX3CR1^{gfp} expression. **b**-**e** Histograms showed percentages of single positive IL-6+, TNF-α+ or double positive IL-6+ TNF-α+ in Ly6C^{low} CX3CR1^{high} monocytes (**b**) or in Ly6C^{high} CX3CR1^{low} monocytes (**c**-**e**) determined by flow cytometry after intracellular cytokine staining. Monocytes were stimulated or not with 300 ng/ml LPS for 4h, alone or in co-cultures with Sca-1+ renal cells (ratio Sca-1+ : monocytes, 1:5; 1:3; 1:2) (n=8 per group). **P<0.01 ***P<0.001 ns, not significant, compared to LPS treated monocytes with Mann-Whitney's U test.

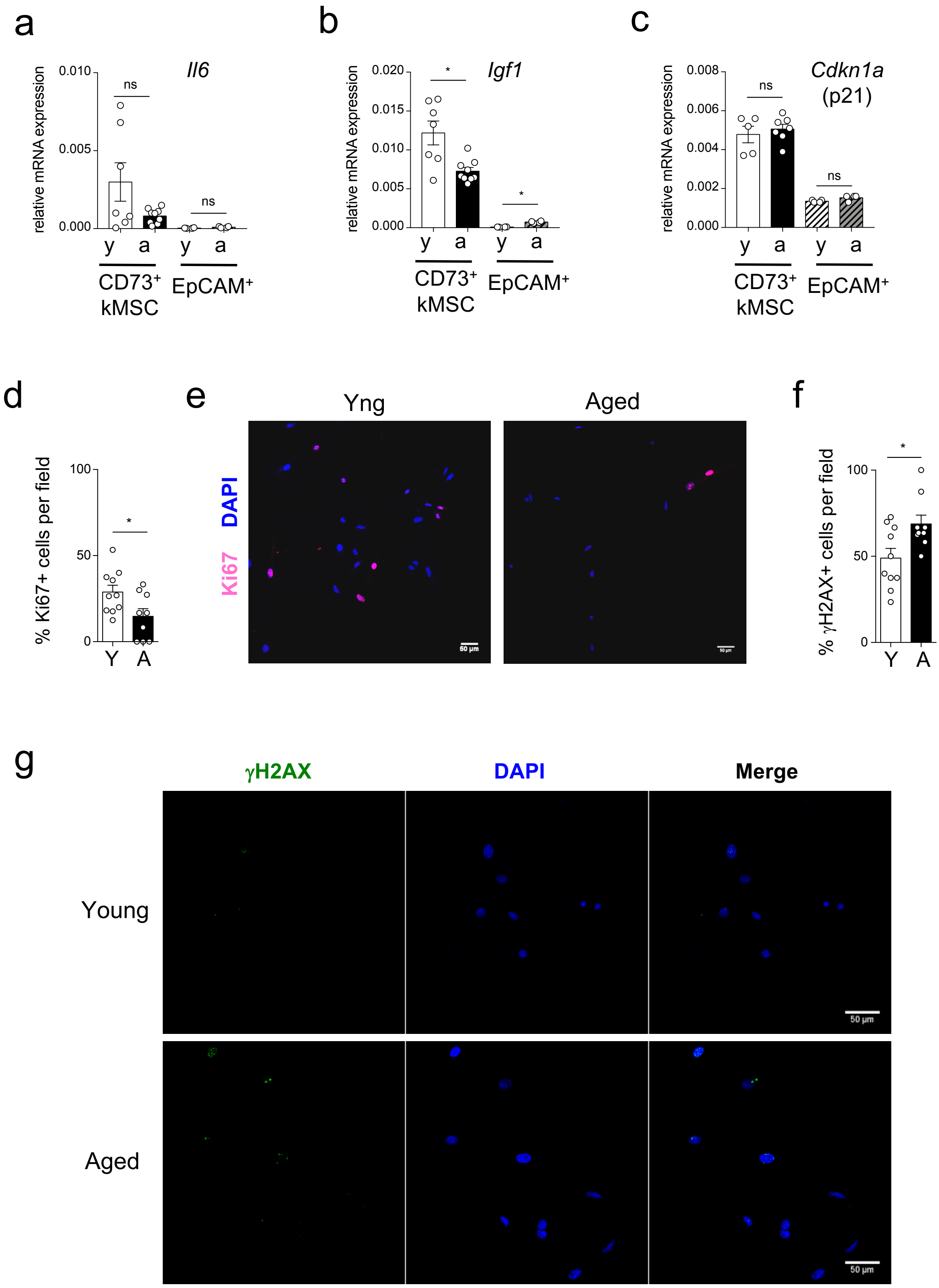

Supplementary Fig. 6 Senescence markers of CD73+ kMSCs and of Sca-1+ epithelial cells.

a-**c** Gene expression of *Il6* (**a**), *Igf1* (**b**), and *Cdkn1a* (p21) (**c**) by young or aged CD73+ kMSCs (n=7-9 per group) and Sca-1+ EpCAM+ epithelial cells (n=4 per group) relative to house-keeping gene. **d**-**g** Percentages of positive cells per field (**d**, **f**) and confocal images (**e**, **g**) of young or aged CD73+ kMSCs stained with anti-Ki67 (**d**, **e**) or anti-γH2AX (**f**, **g**). Nuclei stained with DAPI (blue). Scale bar (50 µm). *P<0.05 **P<0.01 ***P<0.001 ns, not significant, with Mann Whitney t test or Kruskal-Wallis with Dunn's post-test.
