# Supplementary material for: Kidney inflammaging is promoted by CCR2+ macrophages and tissue-derived micro-environmental factors
Source: Cell Mol Life Sci. 2020 Dec 12;78(7):3485–501. doi: 10.1007/s00018-020-03719-0 (PMC8038964; doi:10.1007/s00018-020-03719-0)
Supplement: Supplementary file 4 — Supplementary file4: Supplementary figures (PDF 45 KB) [file 18_2020_3719_MOESM4_ESM.pdf]

Supplementary Informations

Kidney inflammaging is promoted by CCR2+ macrophages and tissue-derived micro-environmental factors

Lefèvre L, Iacovoni JS, Martini H, Bellière J, Maggiorani D, Dutaur M, Marsal DJ, Decaunes P, Pizzinat N, Mialet-Perez J, Cussac D, Parini A and V Douin-Echinard.

Cellular and Molecular Life Sciences

Corresponding authors :
Pr Angelo Parini
angelo.parini@inserm.fr

Dr Victorine Douin-Echinard
victorine.douin@inserm.fr

Institute of Metabolic and Cardiovascular Diseases, UMR1048, 31432 Toulouse, Cedex 4, France
Paul Sabatier University, 31062 Toulouse, Cedex 9, France

Supplementary Methods

Supplementary Tables
Table S1
Table S2

Supplementary Methods

Histochemistry

Kidneys were fixed for 6h in 3.4% PFA then in 70% Ethanol and included in paraffin. Tissue sections were stained with sirius red and digitized with a Hamamatsu NanoZoomer. Stained areas and total surface from each section were determined using color-based thresholding and quantified using Fidji software as previously described (1).

Biochemistry

Creatinin and blood urea nitrogen (BUN) were measured in plasma samples with ABX Pentra reagents using the Pentra C400 analyzer (HORIBA Medical).

References

1. Laroumanie F*, et al.* (2014) CD4+ T cells promote the transition from hypertrophy to heart failure during chronic pressure overload. *Circulation* 129(21):2111-2124.

Table S1. List of the antibodies used for DECyt analysis

name	alternative name	clone	purshased from
**Set A**			
CD45 APC-Cy7		30-F11	Biolegend
Ly6C PB		HK1.4	Biolegend
MHCII FITC	I-A/I-E	M5/114.15.2	Biolegend
CCR2 PE		FAB5538	R&D Systems
CD14 PercPCy5.5		Sa-2-8	eBioscience
CD64 APC	Fc gamma RI	X54-5/7.1	Biolegend
**Set B**			
Sca-1 PB	Ly6A/E	D7	Biolegend
CD31 PercPCy5.5	PECAM1	390	Biolegend
CD45 APC-Cy7		30-F11	Biolegend
CD326 FITC	EpCAM	G8.8	Biolegend
mEFSK4 PE	anti-Feeder Cells	mEF-SK4	Miltenyi Biotec
CD73 PE-Cy7	5’ecto-nucleotidase	TY/11.8	Biolegend
CD140b APC	PDGFRß	APB5	Biolegend

Table S2. Sequences of the primers used for RT-QPCR

gene	Primer sequences (5’ -> 3’)	Ref_Seq
Nt5e	F: CTTCATGAACATCCTGGGCT<br>R: AACGTTTCTGAGGAGGGGAT	NM_011851
Cdkn1c	F: ATCACCAATCAGCCAGCAGAA<br>R: CTACGCGCTATCACTGGGAAG	NM_009876.4
Cdkn2c	F: GGGGGACCTAGAGCAACTTAC<br>R: CTCCGGATTTCCAAGTTTCA	NM_007671.2
Cx3cl1	F: TGGCTTTGCTCATCCGCTATCAG<br>R: CGTCTGTGCTGTGTCGTCTCC	NM_009142.3
Cxcl2	F: CGCTGTCAATGCCTGAAG<br>R: GGCGTCACACTCAAGCTCT	NM_009140.2
Ccl2	F: AAACCTGGATCGGAACCAAAT<br>R: TACGGGTCAACTTCACATTCAAA	NM_011333.3
Il6	F: GAGGATACCACTCCCAACAGACC<br>R: AAGTGCATCATCGTTGTTCATACA	NM_031168
Il1a	F: TTGGTTAAATGACCTGCAACA<br>R: GAGCGCTCACGAACAGTTG	NM_010554.4
Il10	F: TTCAGCCAGGTGAAGACTTTCT<br>R: GCTTGGCAACCCAAGTAACC	NM_010548.2
Tgfb1	F: AGGGCTACCATGCCAACTTCT<br>R: CCGGGTTGTGTTGGTTGTAGA	NM_011577.2
Vegfa	F: CAGCAGATGTGAATGCAGACCAA<br>R: CTTTCTCCGCTCTGAACAAGGC	NM_001025250.3
Cxcl12	F: CTCAACACTCCAAACTGTGCC<br>R: TTGGGCTGTTGTGCTTACTTG	NM_021704.3
Cxcr4	F: CCATGGAACCGATCAGTGTGA<br>R: CAGGGTTCCTTGTTGGAGTCA	NM_009911.3
Igf1	F: GCTCTTCAGTTCGTGTGTGGAC<br>R: AGCCTGTGGGCTTGTTGAAGTA	NM_010512.4
Ccr2	F: CCTTGGGAATGAGTAACTGTGTGA<br>R: AATGACAGGATTAATGCAGCAGTGT	NM_009915.2
Tnfa	F: AGCCGATTTGCTATCTCATACCA<br>R: GGGCTCATACCAGGGTTTGA	NM_013693
Il1b	F: CTGCACTACAGGCTCCGAGAT<br>R: TGTTGGTTGATATTCTGTCCATTG A	NM_008361.4
Il12b	F: CGGACGGTTCACGTGCTC<br>R: CACATGTCACTGCCCGAGAGT	NM_001303244.1

Cdkn2a	F: CCGAACTCTTTCGGTCGTACCC<br>R: CTGCTACGTGAACGTTGCCCA	NM_001040654.1
Rplp0	F: GCTTCATTGTGGGAGCAGAC<br>R: ATGGTGTTCTTGCCCATCAG	NM_007475.5
Trp53	F: TCCTGGCTGTAGGTAGCGACT<br>R: ATCCGACTGTGACTCCTCCAT	NM_001127233.1<br>NM_011640.3
